# The association between physical activity and academic procrastination among college students: the mediating role of self-efficacy and the moderating role of sleep quality

**DOI:** 10.3389/fpsyg.2026.1883617

**Published:** 2026-10-23

**Authors:** Jin Han, Zigu Zhang, Mingyang Liu, Zhijian Rao, Lifang Zheng

**Affiliations:** 1 College of Physical Education, Shanghai University, Shanghai, China; 2 College of Physical Education, Shanghai Normal University, Shanghai, China; 3 Exercise Biological Center, China Institute of Sport Science, Beijing, China

**Keywords:** academic procrastination, moderated mediation model, physical activity, self-efficacy, sleep quality

## Abstract

**Objectives:**

This study aimed to investigate the associations between physical activity and academic procrastination among college students within a cross-sectional framework while examining the mediating role of self-efficacy and the moderating role of sleep quality.

**Methods:**

A cross-sectional survey was conducted with 428 college students from universities in Shanghai. Participants completed the International Physical Activity Questionnaire-Short Form (IPAQ-S), the Academic Procrastination Scale, the General Self-Efficacy Scale (GSES), and the Pittsburgh Sleep Quality Index (PSQI). Data were analyzed using the PROCESS macro in SPSS 26.0 to test a moderated mediation model, adjusting for demographic covariates, including gender, age, academic major, academic year, only-child status, and geographic origin.

**Results:**

(1) Physical activity was significantly and negatively associated with academic procrastination (*β* = −0.127, 95% confidence interval [CI] [−0.223, −0.031], *p* < 0.05) and positively associated with self-efficacy (*β* = 0.200, 95% CI [0.103, 0.296], *p* < 0.001). Self-efficacy was concurrently and negatively associated with procrastination (*β* = −0.235, 95% CI [−0.330, −0.142], *p* < 0.001). (2) Self-efficacy accounted for a significant portion of the association between physical activity and academic procrastination, with a significant indirect effect of −0.047 (95% CI [−0.078, −0.019]), accounting for 27.01% of the total effect. (3) Sleep quality significantly moderated the association between physical activity and self-efficacy (*β* = −0.147, 95% CI [−0.236, −0.056], *p* < 0.01) and the direct association between physical activity and academic procrastination (*β* = 0.147, 95% CI [0.058, 0.236], *p* < 0.01). (4) Conditional process analyses revealed that the indirect effect via self-efficacy and the direct association with procrastination were significant only among students with average or good sleep quality, but not among those with poor sleep quality.

**Conclusion:**

The findings reveal significant concurrent associations among physical activity, self-efficacy, sleep quality, and academic procrastination. Self-efficacy partially accounted for the relationship between physical activity and procrastination. Crucially, sleep quality emerged as a critical boundary condition: the linkages between physical activity and reduced procrastination (both direct and indirect) were evident only when sleep quality was adequate. These results underscore the necessity of adopting a stratified approach in future interventions—targeting physical activity and self-efficacy for adequately rested students, while integrating sleep hygiene support for those with poor sleep—pending validation through longitudinal or experimental designs.

## 1 Introduction

Against the backdrop of intensifying competition in higher education, outstanding academic performance is pivotal for students’ future development. However, academic procrastination—defined as the irrational delay in completing planned learning tasks (Klingsieck, 2013)—is a pervasive phenomenon that significantly undermines students’ learning outcomes and mental health. Research indicates that approximately half of all college students experience situational learning procrastination (Day et al., 2000), which is strongly linked to poorer academic performance and heightened psychological stress (Chen, 2021; Doherty, 2006; Ferrari, 2011). This issue is particularly salient in the Chinese higher education context. Chinese college students face unique pressures from the legacy of the Gaokao-driven examination culture, which fosters a lifelong performance orientation; the rapid expansion of higher education enrollment since 1999 has intensified intra-institution competition for limited graduate school admissions and prestigious employment opportunities; and the widespread phenomenon of “involution” (neijuan) has been empirically linked to elevated academic burnout and reduced self-regulatory capacity (Liu et al., 2024). Among Chinese university students, academic procrastination has been linked to maladaptive perfectionism and fear of failure; empirical studies show that concern over mistakes and fear of failure mediate the links between perfectionism, self-esteem, and procrastination (Wang X. et al., 2026; Zhang et al., 2018). While cross-sectional and longitudinal studies have established associations between procrastination and poorer academic success (Beswick et al., 1988; Park and Sperling, 2012; Tice and Baumeister, 1997), the magnitude of these associations varies substantially across contexts. Notably, isotemporal substitution evidence shows that only moderate-to-vigorous physical activity (MVPA) yields robust benefits, whereas light physical activity (LPA) may show negligible or even paradoxical associations with procrastination (Kuai et al., 2025). These inconsistencies underscore the need to clarify underlying mechanisms and develop targeted intervention strategies.

Traditional interventions often target time management, motivation, or cognitive behavioral correction (Shalev, 2018; Wolters, 2003). Recently, the integration of lifestyle and psychological resources has offered new insights. Regular physical activity, a key non-pharmacological intervention, has been concurrently associated with lower levels of procrastination, potentially by supporting the self-regulating resources depleted through academic stress (Baumeister et al., 2007). Empirical studies consistently report a negative association between physical activity and academic procrastination (Zhuan et al., 2024). However, the mechanisms linking these constructs remain incompletely understood, particularly regarding the role of self-efficacy.

A critical distinction exists between general self-efficacy and domain-specific academic self-efficacy (ASE). ASE reflects beliefs about one’s capability to perform specific academic tasks. In contrast, self-efficacy denotes a broader, generalized sense of competence across diverse challenging situations (Bandura, 1997; Heffernan, 1988). According to social cognitive theory, mastery experiences in one domain (e.g., persisting through strenuous physical activity) can enhance an individual’s generalized self-efficacy, which may subsequently transfer to novel domains such as academic pursuits (Bandura, 1977; McAuley and Blissmer, 2000). Indeed, empirical evidence supports this cross-domain transfer: Ren et al. demonstrated that self-efficacy—not ASE—significantly mediated the relationship between physical activity and academic procrastination among Chinese college students (Ren et al., 2021). Moreover, Klassen et al. found that self-efficacy for self-regulation was the strongest predictor of academic procrastination among various self-beliefs (Klassen et al., 2008). Self-efficacy thus serves as a foundational cognitive construct that is concurrently associated with adaptive coping across contexts. Therefore, self-efficacy may act as a critical mediator through which physical activity exerts its benefits on academic behaviors, operating in parallel with—or as a precursor to—domain-specific academic self-efficacy.

Beyond psychological factors, sleep quality is fundamental to cognitive function and emotional regulation. Good sleep supports memory consolidation, stabilizes mood, and enhances executive function (Hyndych et al., 2025), thereby directly mitigating procrastination tendencies arising from attentional lapses or emotional anxiety (Lund et al., 2010). The interplay between sleep and physical activity is complex: while acute sleep deprivation may impair exercise performance (Craven et al., 2022), meta-analytic evidence confirms that regular physical activity is associated with improved sleep quality (Kredlow et al., 2015). Importantly, sleep quality may act as a regulator in this mechanism. Adequate sleep likely provides the physiological foundation necessary for physical activity to be associated with enhanced self-efficacy; conversely, poor sleep may blunt this benefit. Moreover, good sleep may optimize emotional regulation and cognitive resource reserves, potentially amplifying the protective effect of high self-efficacy against procrastination (Tang and He, 2025). However, whether sleep quality moderates the associations between physical activity, self-efficacy, and academic procrastination remains underexplored.

Furthermore, several sociodemographic characteristics warrant consideration as potential confounders when examining these associations. In the Chinese context, only-child status is particularly relevant: individuals raised without siblings often receive concentrated parental expectations and investment, which may shape their self-efficacy beliefs and coping styles, thereby influencing procrastination tendencies. Similarly, geographic origin (urban vs. rural) reflects systematic differences in access to educational resources, physical activity facilities, and living environments, which can affect both physical activity habits and sleep quality. To isolate the unique contributions of physical activity, self-efficacy, and sleep quality, these variables were included as covariates in the present study.

In summary, although prior studies have separately explored the relationships between physical activity, self-efficacy, sleep quality, and academic procrastination, critical gaps remain. Specifically, no study has simultaneously integrated self-efficacy as a mediator and sleep quality as a moderator within a single moderated mediation framework to examine how physical activity relates to academic procrastination. Moreover, while sleep quality is known to be associated with both physical activity and self-efficacy, its role as a boundary condition—particularly its potential to moderate the physical activity → self-efficacy pathway—has never been empirically tested. Clarifying this complex mechanism holds important theoretical and practical implications for developing tailored, multi-faceted intervention strategies that target modifiable lifestyle factors (physical activity and sleep) and psychological resources (self-efficacy) to mitigate academic procrastination among college students. Thus, the present study makes three novel contributions: (a) it is the first to test a unified moderated mediation model linking physical activity to academic procrastination via self-efficacy, conditioned by sleep quality; (b) it extends prior work by examining whether sleep quality moderates both the direct path (physical activity → procrastination) and the first stage of the indirect path (physical activity → self-efficacy); and (c) it provides the first evidence on whether the beneficial associations between physical activity and self-efficacy and procrastination are contingent upon adequate sleep—a finding that could inform stratified intervention designs. Accordingly, this study establishes a moderated mediation model to examine: (1) whether physical activity is negatively associated with academic procrastination; (2) whether self-efficacy mediates the relationship between physical activity and academic procrastination; and (3) whether sleep quality moderates both the association between physical activity and academic procrastination and the first half of the mediation path (physical activity → self-efficacy).

## 2 Theoretical basis and hypothesis

### 2.1 The relationship between physical activity and academic procrastination

According to self-regulatory resource theory, self-control operates as a limited, depletable resource analogous to a muscle—acts of self-regulation consume this resource, and its depletion manifests as ego depletion and subsequent failures of self-control. Academic procrastination, defined as the irrational delay of planned learning tasks, represents a quintessential failure of self-regulation resulting from depleted regulatory resources (Li et al., 2022; Wei, 2023). Regular physical activity, however, serves as a resource-replenishing behavior: by enhancing physiological functions (e.g., neurotransmitter regulation and cardiovascular efficiency) and fostering positive psychological experiences (e.g., stress relief and mood improvement), exercise increases the pool of available self-regulatory resources, thereby mitigating the propensity to delay academic tasks (Codina et al., 2020; Herzog-Krzywoszanska et al., 2024; Li et al., 2026; Zhang et al., 2026a). This theoretical logic directly supports the hypothesized direct negative association between physical activity and procrastination.

Empirical evidence is consistent with this resource-replenishment mechanism. Kuai et al.’s (2025) research based on the isotemporal substitution model further revealed that, after controlling for demographic variables, both moderate-to-vigorous physical activity (MVPA) and light physical activity (LPA) could significantly and negatively predict academic procrastination among college students. Furthermore, Yang et al. (2022) similarly reported that higher levels of physical activity were associated with fewer procrastination behaviors. Additionally, research using mediation models has confirmed that physical activity not only directly and negatively predicts academic procrastination but also exerts an indirect influence by enhancing psychological variables such as physical self-awareness and self-esteem (Ren et al., 2023). Overall, existing evidence strongly supports the argument that physical activity serves as a protective factor against academic procrastination.

Based on the above theoretical and empirical evidence, this study proposes Hypothesis 1:

*H1*: Physical activity is negatively associated with academic procrastination.

### 2.2 The mediating role of self-efficacy

While self-regulatory resource theory explains the direct resource-replenishing effect of physical activity, social cognitive theory (Bandura, 1977) provides the theoretical logic for how these resources are translated into academic behavior via self-efficacy. According to this theory, an individual’s belief in their capability to successfully execute tasks—self-efficacy—is derived from four principal sources: performance accomplishments (mastery experiences), vicarious experience, verbal persuasion, and physiological states. Physical activity serves as a powerful mastery experience: successfully engaging in and sustaining exercise builds a generalized sense of competence and self-confidence (McAuley and Blissmer, 2000). This enhanced self-efficacy then transfers to the academic domain, increasing students’ confidence in tackling challenging coursework, enabling greater proactive coping, and reducing avoidance behaviors driven by fear of failure. This theoretical chain—physical activity → enhanced self-efficacy → reduced procrastination—establishes self-efficacy as the psychological mechanism through which the regulatory resources generated by exercise are converted into academic behavioral change.

Self-efficacy refers to an individual’s confidence and belief in their capability to successfully complete a specific task or achieve a goal (Waddington, 2023). Social cognitive theory further specifies that self-efficacy influences not only behavioral choices but also the level of effort and perseverance in the face of difficulties (Schunk and Hanson, 1989). Students with high self-efficacy demonstrate greater perseverance and effort in their studies and exhibit better academic self-control, which helps avoid procrastination; conversely, low self-efficacy is linked to self-doubt, anxiety, and task avoidance, leading to procrastination (Jing et al., 2025; Santos et al., 2025). In the academic field, students with high self-efficacy typically exhibit lower levels of academic procrastination. This theoretical logic directly supports the hypothesized mediating role of self-efficacy.

Empirical support for this mediated pathway is well-established. Li et al. (2022) found that self-efficacy and self-control acted as parallel mediators between physical activity and procrastination among Chinese university students. Ren et al. (2021) specifically identified self-efficacy as a significant mediator in the relationship between physical activity and academic procrastination. Gao et al. (2025) further demonstrated that physical exercise significantly positively correlates with academic self-efficacy among college students.

Based on the above theoretical analysis, this study proposes Hypothesis 2:

*H2*: Self-efficacy mediates the relationship between physical activity and academic procrastination. That is, higher physical activity is associated with greater self-efficacy, which in turn is associated with lower academic procrastination.

### 2.3 The moderating role of sleep quality

The extent to which physical activity can generate regulatory resources and bolster self-efficacy is contingent upon the individual’s capacity to preserve and utilize those resources—a proposition explained by conservation of resources (COR) theory (Hobfoll, 1989). Within this framework, sleep quality is conceptualized not only as a resource generated by physical activity but also as a fundamental resource reservoir that determines whether the psychological benefits of exercise (e.g., enhanced self-efficacy) can be effectively realized. Adequate sleep provides the cognitive and physiological foundation necessary for translating mastery experiences from physical activity into self-efficacy beliefs, whereas poor sleep depletes this foundation regardless of activity level. This logic positions sleep quality as a moderator that amplifies or attenuates the effects of physical activity, rather than as a simple transmission mechanism. We acknowledge that alternative specifications (e.g., sleep quality as a mediator) are plausible and have been tested elsewhere (Wang G. et al., 2026; Wang X. et al., 2026); however, given our focus on identifying boundary conditions for the physical activity–procrastination relationship, the moderating framework offers a more direct test of the conditions under which physical activity confers its benefits. COR theory posits that individuals strive to acquire, retain, and protect their valued resources and that the potential or actual loss of these resources is fundamentally threatening. Within this framework, physical activity represents an investment behavior that generates physiological and psychological resources (e.g., energy, positive emotions, and self-efficacy), while good sleep quality is a key process for restoring and conserving these resources (Wang G. et al., 2026). When sleep quality is adequate, the resources acquired through physical activity are effectively preserved and amplified, allowing the self-efficacy-enhancing and procrastination-reducing benefits of exercise to be fully realized (Kuai et al., 2025). Conversely, poor sleep quality depletes the resource reservoir, undermining the individual’s capacity to utilize the resources generated by physical activity, thereby attenuating or nullifying the beneficial effects of exercise.

Physical activity provides mastery experiences—successful regulation of exercise intensity, persistence through fatigue, and achievement of fitness milestones—which serve as the primary source of self-efficacy (Bandura, 1977). However, the internalization of these mastery experiences into self-efficacy beliefs requires intact cognitive and emotional processing resources. Sleep quality, as the principal mechanism for restoring these resources, determines whether and to what extent the mastery experiences from exercise can be cognitively encoded and emotionally consolidated into self-efficacy. When sleep quality is high, the restored executive functions and emotional regulation capacity allow individuals to fully appraise and integrate their exercise mastery experiences, thereby strengthening self-efficacy; when sleep quality is poor, impaired cognitive processing hinders this translation, leaving the self-efficacy-enhancing potential of physical activity unrealized. This theoretical logic establishes sleep quality not only as a correlate of self-efficacy but also as a boundary condition that governs the physical activity → self-efficacy conversion pathway.

This theoretical proposition is empirically supported by recent large-scale evidence. Wang G. et al. (2026) investigated 8,899 Chinese college students and identified a significant chain mediation pathway in which exercise adherence improved sleep quality, which subsequently enhanced self-efficacy. This finding provides direct evidence that sleep quality functions as a critical precursor in the translation of physical activity benefits into self-efficacy, lending strong empirical support to our hypothesized moderating role. Complementarily, Zhang et al. (2026b) demonstrated in a sample of 10,970 college students that physical exercise indirectly optimizes sleep quality through the synergistic mechanism of enhanced self-efficacy and emotional control, revealing a dynamic, bidirectional relationship between physical activity, self-efficacy, and sleep quality.

Complementing COR theory, the cognitive energy restoration perspective provides the micro-mechanistic explanation for why sleep quality moderates these pathways. A growing body of research indicates that sleep plays a vital role in cognitive functioning, with significant negative correlations between poor sleep quality and performance across critical cognitive domains, including attention, memory, and executive functions (Kaczmarek et al., 2025; Santos et al., 2025). When sleep quality is good, the cognitive energy restored during sleep allows the psychological benefits derived from physical activity (e.g., enhanced self-efficacy) to be fully translated into academic confidence and proactive behavior (Schmitt et al., 2017). Conversely, sleep loss leads to resource depletion and impaired cognitive energy, indicating that even if an individual maintains physical activity, the psychological benefits may be difficult to utilize effectively, greatly reducing the impact on self-efficacy and procrastination reduction.

Together, COR theory and the cognitive energy restoration perspective provide the theoretical rationale for why sleep quality moderates both the direct path (physical activity → procrastination) and the first stage of the mediation path (physical activity → self-efficacy). This integrative logic is consistent with emerging empirical evidence: Kuai et al. (2025) reported that higher sleep quality enhances (i.e., positively moderates) the effect of replacing sedentary behavior with physical activity on reducing procrastination. This suggests that when individuals experience good sleep (ensuring adequate resource conservation), the resource gains from physical activities can be more effectively transformed into the behavioral execution capability needed to combat procrastination.

While alternative specifications (e.g., sleep quality as a mediator of the physical activity–self-efficacy link) are plausible, the bidirectional association between physical activity and sleep quality has been documented in recent large-scale work. Supporting this bidirectionality, research has found that earlier bedtimes or sufficient sleep duration are associated with increased levels of moderate-to-vigorous physical activity (MVPA) and total physical activity (PA) duration the following day (Leota et al., 2025). On the other hand, regular physical activity, especially leisure-time aerobic exercise, has been widely proven to effectively improve sleep quality (Huang et al., 2023; Reid et al., 2010; Xie et al., 2021). The bidirectional association between physical activity and sleep quality aligns with our conceptualization of sleep quality as a boundary condition that amplifies the returns on physical activity investment, consistent with COR theory’s emphasis on resource protection rather than unidirectional transmission.

In summary, by integrating COR theory, the cognitive energy restoration perspective, and specific empirical evidence, we conceptualize sleep quality not as a mere antecedent or outcome but as a pivotal moderator that governs both the direct effect of physical activity on procrastination and the indirect effect via self-efficacy.

Based on this integrative reasoning, we propose Hypothesis 3:

*H3*: Sleep quality moderates the path through which physical activity affects academic procrastination. Specifically:

*H3a*: Sleep quality moderates the association between physical activity and academic procrastination. Under conditions of high sleep quality, the negative association between physical activity and academic procrastination is stronger.

*H3b*: Sleep quality moderates the first half of the mediation pathway (physical activity → self-efficacy). The positive association between physical activity and self-efficacy is more evident or stronger when sleep quality is high, thereby influencing the overall indirect effect on academic procrastination (see Figure 1).

**Figure 1:**
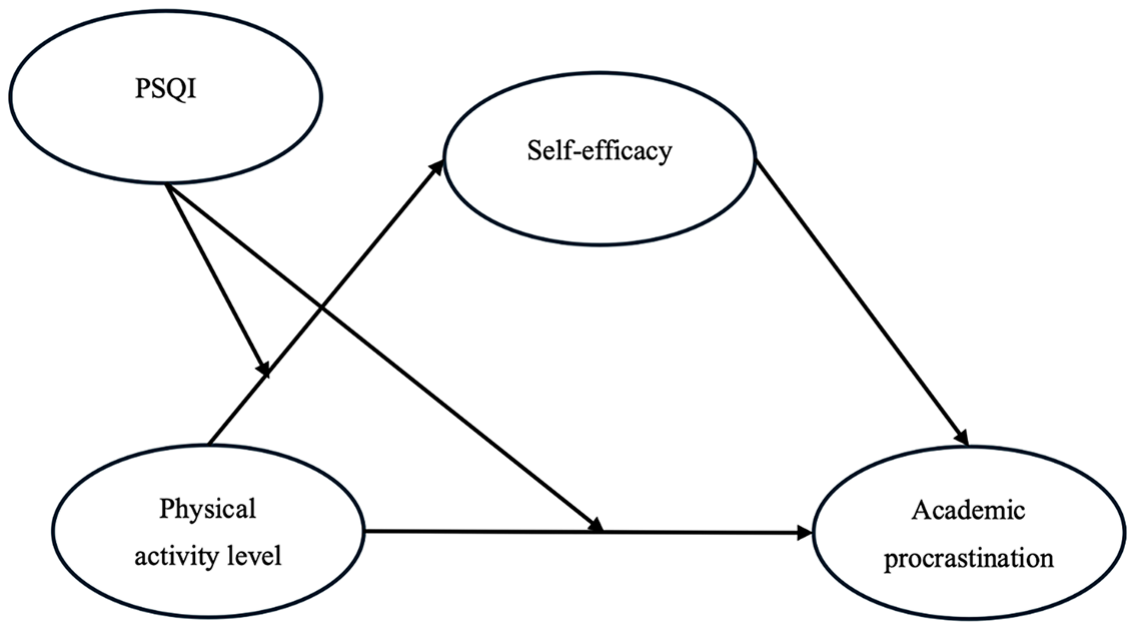
A hypothesis chain mediation model of physical activity and academic procrastination.

## 3 Materials and methods

### 3.1 Study design and participants

This cross-sectional study was conducted at three selected universities in Shanghai, China. The study protocol was approved by the Ethics Review Committee of Shanghai University (ECSHU2025-071), and all procedures followed the ethical guidelines of the Declaration of Helsinki. All participants provided informed consent prior to the survey and received a small gift upon completion.

The following inclusion and exclusion criteria were applied. The inclusion criteria were as follows: (1) full-time undergraduate or postgraduate students aged 18–26 years enrolled in the three selected universities in Shanghai and (2) no cognitive impairment preventing independent completion of the online questionnaire. The exclusion criteria were as follows: (1) fourth-year undergraduates (due to low on-campus availability during the survey period); (2) diagnosed sleep disorders (e.g., insomnia disorder and obstructive sleep apnea–hypopnea syndrome) or medical conditions affecting physical activity capacity (e.g., musculoskeletal injuries and cardiovascular diseases); and (3) regular use of medications impacting sleep or motor function. Additionally, to ensure data validity, participants with incomplete questionnaire responses (missing >20% of items) or excessively short completion times (<5 min) were excluded during data cleaning.

The three universities were purposively selected to represent the diversity of regional higher education ecosystems: (1) Shanghai University, a comprehensive “Double First-Class” and 211-project national university; (2) Shanghai Normal University, a key institution specializing in teacher education and liberal arts; and (3) Shanghai Polytechnic University, a vocational-technical university emphasizing applied sciences and engineering. This selection ensured variations in academic culture, student demographics, and curricular focus. Within each institution, stratified random sampling technique was performed based on three predefined strata: academic year (freshman, sophomore, junior, and postgraduate), major category (liberal arts, science, and engineering), and gender. Using class-specific enrollment rosters—pre-compiled by the academic affairs offices of each university and organized by grade, major, and class cohort—as the primary sampling frames, we defined each class as the primary sampling unit and randomly selected target classes within each stratum via a computer-generated random number sequence. All students within the selected classes were invited to participate via official class communication channels (e.g., university-approved WeChat groups).

An *a priori* sample size justification was conducted using GPower 3.1. For a multiple regression analysis incorporating 10 predictors, a medium effect size (*f*^2^ = 0.15), *α* = 0.05, and power = 0.95, the minimum sample size was calculated to be 89. Given that interaction effects (moderation) typically require larger samples, we additionally evaluated the achieved power for the full model (*R*^2^ deviation from zero). With the final sample of *N* = 428, the study attained an actual power exceeding 0.99 for detecting the overall model effect. Although the minimum sample size required for the overall model test was 172, our sample size of 428 far exceeds this threshold, providing robust statistical power for testing the hypothesized moderated mediation model. To account for potential invalid responses, we initially aimed to recruit 150 participants per university, totaling 450 questionnaires.

A total of 450 questionnaires were initially distributed (*n* = 150 per university). During data cleaning, items with missing rates below 5% across the full sample were retained, while participants with more than 20% missing items were excluded. To identify careless responding, the online survey platform was programmed to automatically reject submissions with total completion times below 5 min, and responses displaying implausible patterns (e.g., identical answers to all items and logically inconsistent sequences) were manually identified and excluded. After applying the exclusion criteria, 428 valid samples were retained, yielding an effective response rate of 95.11%. The response rates for Shanghai University, Shanghai Normal University, and Shanghai Polytechnic University were 94.67% (142/150), 96.00% (144/150), and 94.67% (142/150), respectively. The age of the valid samples ranged from 18 to 26 years. The sample composition is as follows: By grade, there were 120 freshmen (28.0%), 129 sophomores (30.1%), 92 juniors (21.5%), and 87 postgraduates (20.3%). By geographic origin, there were 121 students from rural areas (28.3%) and 307 students from urban areas (71.7%). By major category, there were 182 students (42.5%) majoring in liberal arts and 246 students (57.5%) majoring in science and engineering.

### 3.2 Research tools

#### 3.2.1 International Physical Activity Questionnaire-Short Form (IPAQ-S)

The International Physical Activity Questionnaire-Short Form (IPAQ-S) was used to assess participants’ total physical activity level over the past 7 days (Craig et al., 2003). This scale consists of seven items, with six core items, respectively, querying the frequency (days/week) and average daily duration (minutes/day) of walking, moderate-intensity activity, and vigorous-intensity physical activity. For example, participants were asked: “In the past 7 days, on how many days did you engage in vigorous physical activity, such as heavy lifting, digging, aerobics, or fast bicycling?” Metabolic equivalent of task (MET) values were assigned as follows: 3.3 METs for walking, 4.0 METs for moderate-intensity activity, and 8.0 METs for vigorous-intensity activity. The total weekly physical activities (in MET-minutes/week) were calculated as follows: walking (W) = 3.3 × minutes of walking × days; moderate physical activity (MPA) = 4.0 × minutes of MPA × days; and vigorous physical activity (VPA) = 8.0 × minutes of VPA × days. The total physical activity (IPA) = W + MPA + VPA.

All IPAQ data cleaning procedures strictly followed the official IPAQ scoring guidelines (IPAQ Research Committee, 2005): (1) weekly frequency of each activity type was truncated to a maximum of 7 days; (2) daily duration of walking, MPA, and VPA was truncated to a maximum of 180 min per day; (3) total weekly MET-minutes exceeding 33,600 were truncated to this upper limit; and (4) responses reporting 0 activity frequency but >0 duration were corrected to 0 duration.

The Chinese version of the IPAQ-S has demonstrated acceptable criterion validity against accelerometer data in Chinese college students (Wang et al., 2025). As a behavioral recall inventory rather than an attitudinal latent construct scale, composite reliability (CR) and average variance extracted (AVE) are not theoretically applicable; internal consistency was therefore evaluated using Cronbach’s *α*, which was 0.71—within the acceptable range for self-reported physical activity measures. Exploratory factor analysis (EFA) with parallel analysis supported a single-factor solution, which explained 57.8% of the variance (KMO = 0.722; Bartlett’s *χ*^2^ = 672.558, *p* < 0.001). Raw MET-minute scores were log (1 + *x*) transformed to reduce positive skewness prior to inclusion in the structural models.

#### 3.2.2 Academic Procrastination Scale

The degree of academic procrastination was assessed using the college Students’ Academic Procrastination Questionnaire compiled by Han Guining (Han, 2008). This questionnaire consists of 26 items rated on a 5-point Likert scale (1 = “never like this” to 5 = “always like this”). A sample item is: “The study plan I make before exams often cannot be completed on time.” Items requiring reverse scoring were first recoded, and then a total score was calculated by summing all items, with higher scores indicating a more severe tendency toward academic procrastination. Prior validation studies have confirmed that this Chinese adaptation has stable reliability and validity in domestic undergraduate populations (Han, 2008). In this study, the Cronbach’s *α* coefficient of this scale was 0.85, indicating good internal consistency reliability. EFA supported the original three-factor structure, accounting for 58.6% of the variance (KMO = 0.918, Bartlett’s test *χ*^2^ = 6734.166, *p* < 0.001). CR was 0.941, and AVE was 0.521, indicating robust convergent validity.

#### 3.2.3 Self-Efficacy Scale

Self-efficacy was measured using the Chinese version of the General Self-Efficacy Scale (GSES) revised by Zhang and Schwarzer (1995). This scale includes 10 items rated on a 4-point scale (1 = “completely incorrect” to 4 = “completely correct”). A representative item is: “I can always manage to solve difficult problems if I try hard enough”. Participants rated each item based on their own situation. A total score was obtained by summing all items, with higher scores indicating a higher level of general self-efficacy. The Chinese version of the Self-Efficacy Scale is widely validated with excellent psychometric properties in Chinese college students (Zeng et al., 2022). In this study, the Cronbach’s *α* coefficient of this scale was 0.917. EFA confirmed the unidimensional structure, accounting for 61.0% of the variance (KMO = 0.928, Bartlett’s test *χ*^2^ = 2908.282, *p* < 0.001). CR was 0.928, and AVE was 0.571, demonstrating excellent convergent validity.

#### 3.2.4 Pittsburgh Sleep Quality Index (PSQI)

The Pittsburgh Sleep Quality Index (PSQI) was used to evaluate participants’ sleep quality over the past month (Buysse et al., 1989). The PSQI comprises 18 self-rated items, summarized into 7 dimensions: subjective sleep quality, time to fall asleep, sleep duration, sleep efficiency, sleep disorders, use of sleep medication, and daytime dysfunction. For example, Item 1 asks: “During the past month, how long (in minutes) has it usually taken you to fall asleep each night?” Each dimension is scored from 0 to 3 according to specific criteria. The sum of the seven dimensions yields the total PSQI score, ranging from 0 to 21, with higher scores indicating poorer sleep quality. The Chinese version of the PSQI has been rigorously validated in Chinese young adult populations, with good cross-cultural equivalence (Zheng et al., 2016). In this study, Cronbach’s *α* = 0.768. EFA supported a three-factor structure (global sleep quality, sleep disturbances, and daily dysfunction), accounting for 72.33% of the variance (KMO = 0.800, Bartlett’s test *χ*^2^ = 1469.333, *p* < 0.001). CR was 0.725, indicating good internal consistency; AVE was 0.527, meeting the conventional 0.50 threshold for convergent validity. The slightly lower KMO value is consistent with prior validation studies, given the PSQI’s heterogeneous components (e.g., medication use, sleep latency, and duration) rather than a unidimensional trait.

### 3.3 Statistical analysis

Statistical analyses were performed using IBM SPSS Statistics 26.0 and the PROCESS macro (Version 4.2). After excluding cases with more than 10% missing responses or straight-line patterns, measurement properties were evaluated through the KMO test and Bartlett’s test of sphericity (construct validity), Cronbach’s *α* (internal consistency), Harman’s single-factor test and collinearity assessment using variance inflation factors (VIFs) assessment (common method bias [CMB]), and skewness/kurtosis (normality). We performed comprehensive regression assumption diagnostics (RESET, Durbin-Watson statistics, VIF, Cook’s distance, studentized residuals, and Breusch–Pagan test); mild heteroscedasticity detected in three models led to the adoption of HC3 robust standard errors. Descriptive statistics and Pearson’s correlations were computed, followed by hierarchical multiple regression models that were adjusted for six covariates (gender, age, academic major, academic year, only-child status, and geographic origin). For hierarchical multiple regression models, continuous predictors were mean-centered; for PROCESS analyses, all continuous predictors were standardized. PROCESS Model 4 was used to test the mediating role of self-efficacy, and PROCESS Model 8 tested the moderated mediation model, in which sleep quality (PSQI) moderates both the physical activity → self-efficacy path and the direct physical activity → academic procrastination path. Bias-corrected 95% CIs based on 5,000 bootstrap resamples were used for inference. Conditional effects were examined at PSQI = −1 standard deviation (SD), mean, and +1 SD. Effects were considered significant if the 95% CIs excluded zero.

## 4 Results

### 4.1 Preliminary diagnostics and regression assumption testing

Preliminary diagnostics indicated that the core assumptions underlying all regression models were sufficiently met to support valid statistical inference (Table 1). Linearity was supported across models, as indicated by non-significant RESET tests (*p*s > 0.05). Standardized residuals approximated a normal distribution, with skewness and kurtosis values below the thresholds of |3| and 10, respectively. Independence of residuals was confirmed by Durbin–Watson statistics ranging from 1.74 to 2.03. Multicollinearity was not detected; variance inflation factors (VIFs) ranged from 1.06 to 4.03, and tolerance values exceeded 0.24, both within acceptable limits (well below the conventional VIF cutoff of 10 and above the corresponding tolerance threshold of 0.10). No unduly influential observations were identified: Cook’s distance values were all below 0.09, and leverage values remained below the 3(*k* + 1)/*n* threshold. Additionally, studentized residuals were examined, with no absolute values exceeding |3|.

**Table 1:** Results of basic assumption tests for the regression models.

Model	RESET *p*	Residual skewness/Kurtosis	BP *p*	DW	Maximum VIF/minimum tolerance	Maximum Cook’s *D*/maximum leverage
Model 1	0.620	−0.205/2.675	<0.001	2.034	4.025/0.248	0.022/0.053
Model 2	0.378	−0.001/4.143	0.093	1.744	4.025/0.248	0.085/0.060
Model 3	0.644	−0.136/2.716	<0.001	2.007	4.031/0.248	0.028/0.065
Model 4	0.379	0.009/4.243	0.029	1.784	4.031/0.248	0.074/0.072

Diagnostics were performed prior to hypothesis testing. HC3 heteroscedasticity-robust standard errors and 5,000 bootstrap confidence intervals were applied in all subsequent analyses due to mild heteroscedasticity detected in Models 1, 3, and 4.

The Breusch–Pagan test revealed homoscedasticity in the academic procrastination outcome model (Model 2, *p* = 0.093). Mild heteroscedasticity was detected in the remaining models (Models 1, 3, and 4; *p*s < 0.05). Consequently, HC3 heteroscedasticity-robust standard errors and bias-corrected 95% confidence intervals based on 5,000 bootstrap resamples were adopted for all regression, mediation, and moderated mediation analyses. Sensitivity analyses excluding cases with studentized residuals exceeding |3| produced substantively identical effect sizes and significance levels, confirming the robustness of the findings.

### 4.2 Common method bias test

All data in this study were collected through student self-reports, which raises concerns regarding common method bias (CMB). To address this, we used a multi-method approach beyond the traditional Harman’s single-factor test. First, we performed Harman’s single-factor test as an initial check. An exploratory factor analysis with unrotated principal components extraction was performed on all measurement items using SPSS26.0. The results revealed 13 factors with eigenvalues greater than 1. Critically, the variance explained by the first factor was 17.693%, which is substantially below the critical threshold of 40% (Podsakoff et al., 2003; Zhou and Long, 2004), indicating that common method bias was not a serious concern in this study. Second, to address the limitations of Harman’s test, we performed a full collinearity test using VIF. This method examines whether variance inflation is induced by method effects rather than substantive theoretical relationships. We specified a model in which each of the four core variables—physical activity, academic procrastination, sleep quality (PSQI), and self-efficacy—was regressed onto all other variables. The resulting VIF values for physical activity, academic procrastination, sleep quality, and self-efficacy were 1.058, 1.116, 1.079, and 1.126, respectively. All values were substantially lower than the conservative cutoff of 3.3 (Kock, 2015), indicating that multicollinearity stemming from common method variance was not a concern in this dataset. Finally, we assessed the normality of the key variables by examining skewness and kurtosis. Consistent with Kline’s (2016) recommendation for structural equation modeling, data can be considered approximately univariate normal if the absolute values of skewness are less than 3 and kurtosis less than 10. The absolute values of skewness and kurtosis for all major variables in this study fell within these thresholds, suggesting that the data were approximately normally distributed and suitable for subsequent parametric analyses.

### 4.3 Descriptive statistics and correlations

Means, standard deviations (SDs), and intercorrelations for all main variables are presented in Table 2. Academic procrastination was significantly negatively correlated with physical activity level (*r* = −0.152, *p* < 0.01), indicating that students who engage in higher levels of physical activity tend to exhibit lower levels of academic procrastination. Academic procrastination was significantly positively correlated with the PSQI (*r* = 0.222, *p* < 0.001), suggesting that students with higher procrastination tendencies tend to report poorer sleep quality. Conversely, academic procrastination was significantly negatively correlated with self-efficacy (*r* = −0.255, *p* < 0.001), implying that students who procrastinate more academically generally possess lower levels of self-efficacy. Regarding the relationship between physical activity and sleep, physical activity level was significantly negatively correlated with the PSQI (*r* = −0.102, *p* < 0.05), indicating that students who are more physically active tend to have better sleep quality. Additionally, physical activity level was significantly positively correlated with self-efficacy (*r* = 0.205, *p* < 0.001), indicating that physically active students tend to have stronger self-efficacy beliefs. Finally, the PSQI was significantly negatively correlated with self-efficacy (*r* = −0.200, *p* < 0.001), suggesting that students with poorer sleep quality tend to report lower self-efficacy. Overall, the results indicate significant interrelationships among the study variables, supporting the proposed associations for the subsequent moderated mediation analysis.

**Table 2:** Correlation analysis of academic procrastination, physical activity level, PSQI, and self-efficacy.

Variable	*M*	SD	Academic procrastination	Physical activity level	PSQI	Self-efficacy
Academic procrastination	3.164	0.490	1			
Physical activity level	1.893	0.800	−0.152** (−0.248, −0.056)	1		
PSQI	8.428	3.291	0.222*** (0.123, 0.319)	−0.102* (−0.194, −0.010)	1	
Self-efficacy	2.788	0.642	−0.255*** (−0.355, −0.143)	0.205*** (0.114, 0.300)	−0.200*** (−0.298, −0.103)	1

*p < 0.05; **p < 0.01; ***p < 0.001.

### 4.4 Regression effect analysis

To further examine the associations between physical activity level, self-efficacy, and academic procrastination, hierarchical regression analyses were performed with self-efficacy and academic procrastination as the dependent variables, respectively, after controlling for the demographic variables of gender, age, academic major, academic year, only-child status, and geographic origin. The results are presented in Table 3.

**Table 3:** Regression effect analysis of physical activity level, self-efficacy, and academic procrastination.

Result variable	Predictor variable	*R*	*R* ^2^	*F*	*β*	*t*	95% CI for *β* LLCI	95% CI for *β* ULCI
Self-efficacy	Gender	0.230	0.053	3.339**	−0.022	−0.445	−0.117	0.074
	Age				0.09	0.945	−0.097	0.276
	Academic year				−0.004	−0.04	−0.172	0.166
	Academic major				−0.036	−0.733	−0.131	0.060
	Only-child status				0.012	0.228	−0.093	0.118
	Geographic origin				−0.041	−0.787	−0.142	0.061
	Physical activity level				0.200***	4.077	0.103	0.296
Academic procrastination	Gender	0.312	0.098	5.666***	−0.016	−0.334	−0.107	0.076
	Age				0.246**	2.648	0.063	0.427
	Academic year				−0.285**	−3.062	−0.468	−0.102
	Academic major				−0.018	−0.371	−0.111	0.076
	Only-child status				0.057	1.122	−0.043	0.157
	Geographic origin				0.006	0.121	−0.087	0.099
	Physical activity level				−0.127*	−2.587	−0.223	−0.031
	Self-efficacy				−0.235***	−4.931	−0.330	−0.142

*p < 0.05; **p < 0.01; ***p < 0.001. *β* denotes the standardized regression coefficient; the 95% confidence intervals are reported on the *β*.

When self-efficacy was used as the outcome variable, the overall model was statistically significant, *R* = 0.230, *R*^2^ = 0.053, *F* = 3.339, *p* < 0.01. The results indicated that physical activity level was significantly positively associated with self-efficacy (*β* = 0.200, *t* = 4.077, *p* < 0.001, 95% CI [0.103, 0.296]), suggesting that higher levels of physical activity were associated with greater self-efficacy among college students. Consistent with the model results, none of the demographic covariates—gender, age, academic major, academic year, only-child status, and geographic origin—were significantly associated with self-efficacy (*p* > 0.05).

When academic procrastination was used as the outcome variable, the overall model was also significant, *R* = 0.312, *R*^2^ = 0.098, *F* = 5.666, *p* < 0.001. After controlling for covariates, age was significantly positively associated with academic procrastination (*β* = 0.246, *t* = 2.648, *p* < 0.01, 95% CI = [0.063, 0.427]), whereas academic year was significantly negatively associated with academic procrastination (*β* = −0.285, *t* = −3.062, *p* < 0.01, 95% CI = [−0.468, −0.102]). In addition, physical activity level was significantly negatively associated with academic procrastination (*β* = −0.127, *t* = −2.587, *p* < 0.05, 95% CI [−0.223, −0.031]), indicating that students with higher physical activity levels tended to report lower academic procrastination. Furthermore, self-efficacy was significantly negatively associated with academic procrastination (*β* = −0.235, *t* = −4.931, *p* < 0.001, 95% CI [−0.330, −0.142]), such that higher self-efficacy was associated with lower levels of academic procrastination. The remaining demographic variables, including gender, only-child status, and geographic origin, were not significantly associated with academic procrastination (*p* > 0.05).

### 4.5 Analysis of mediating role

Building on the regression analysis and following the same analytical approach, this study used PROCESS Model 4 in SPSS to examine the mediating role of self-efficacy in the association between physical activity level and academic procrastination while adjusting for gender, age, academic major, academic year, only-child status, and geographic origin. Bias-corrected 95% confidence intervals were constructed using 5,000 bootstrap resamples. The results are shown in Table 4 and Figure 2.

**Table 4:** Standardized mediation analysis of the association between physical activity and academic procrastination.

Pathway		*β*	Boot SE	95% CI boot LLCI	95% CI boot ULCI	Relative effect proportion
Direct effects	Physical activity → academic procrastination	−0.127	0.049	−0.223	−0.031	72.99%
Indirect effects	Physical activity → self-efficacy → academic procrastination	−0.047	0.015	−0.078	−0.019	27.01%
Total effects	Physical activity → academic procrastination	−0.174	0.049	−0.272	−0.077	100.00%

**Figure 2:**
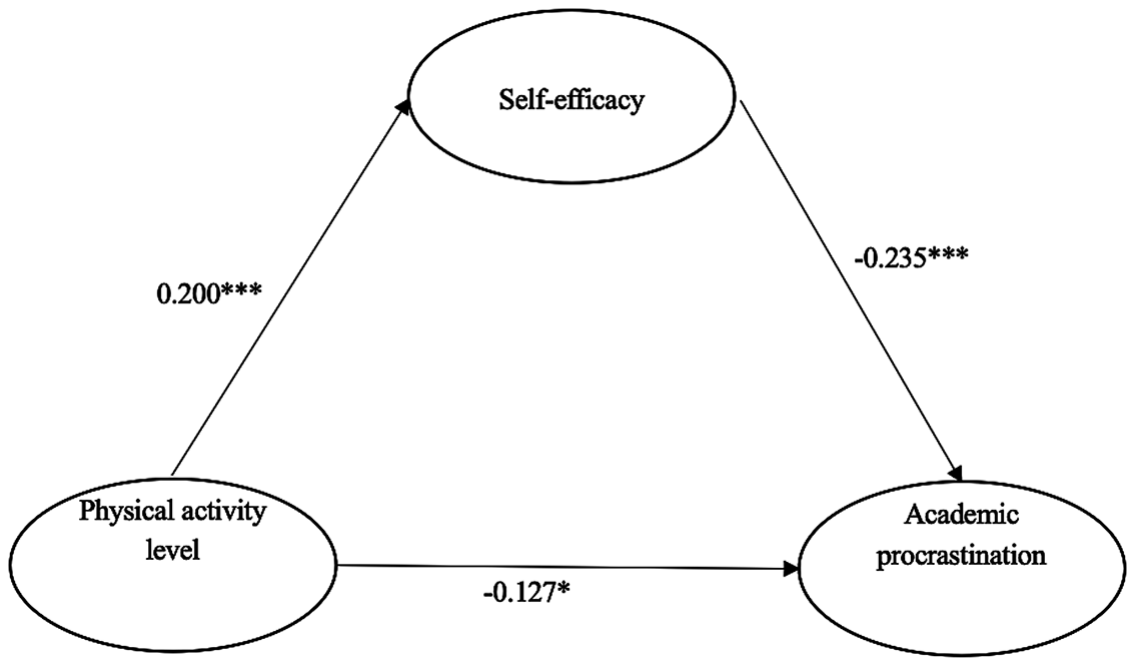
Schematic diagram of the mediation model. The model illustrates the potential mediating role of self-efficacy in the association between physical activity and academic procrastination. Path coefficients are standardized (*p < 0.05; **p < 0.01; ***p < 0.001). The 95% confidence interval for the indirect effect was obtained through bootstrapping (5,000 resamples).

As shown, the total association between physical activity and academic procrastination was significant (*β* = −0.174, Boot SE = 0.049, 95% CI [−0.272, −0.077]) after covariate adjustment. When self-efficacy was included as a mediator, the direct association between physical activity and academic procrastination remained significant (*β* = −0.127, Boot SE = 0.049, 95% CI [−0.223, −0.031]), accounting for 72.99% of the total effect. Furthermore, the indirect association between physical activity and academic procrastination through self-efficacy was also significant (*β* = −0.047, Boot SE = 0.015, 95% CI [−0.078, −0.019]), accounting for 27.01% of the total effect. These findings indicate that self-efficacy partially accounts for the association between physical activity and academic procrastination (i.e., a partial mediation effect was observed).

### 4.6 Moderated mediation analysis

To examine whether sleep quality moderated the associations between physical activity, self-efficacy, and academic procrastination, PROCESS Model 8 was applied to estimate (a) moderation of the path linking physical activity to self-efficacy, and (b) moderation of the direct path linking physical activity to academic procrastination. All continuous predictors were mean-centered prior to creating interaction terms. Standardized coefficients, conditional indirect effects, and the index of moderated mediation are reported below.

#### 4.6.1 Moderated mediation model

As shown in Table 5, when self-efficacy served as the dependent variable, the overall model accounted for a significant proportion of variance (*R*^2^ = 0.108, *F* = 5.626, *p* < 0.001). Physical activity level exhibited a significant positive association with self-efficacy (*β* = 0.179, *t* = 3.718, *p* < 0.001, 95% CI [0.085, 0.273]), whereas the PSQI was significantly and negatively associated with self-efficacy (*β* = −0.190, *t* = −4.049, *p* < 0.001, 95% CI [−0.282, −0.097]). Crucially, the interaction between physical activity and the PSQI was significantly associated with self-efficacy (*β* = −0.147, *t* = −3.169, *p* < 0.01, 95% CI [−0.236, −0.056]), indicating that the positive association between physical activity and self-efficacy was stronger at lower PSQI levels (better sleep) and weaker at higher PSQI levels (poorer sleep).

**Table 5:** Standardized regression analysis of the moderated mediation model.

Outcome	Predictor	*R*	*R* ^2^	*F*	*β*	*t*	95% CI for *β* LLCI	95% CI for *β* ULCI
Self-efficacy	Gender	0.329	0.108	5.626***	−0.046	−0.931	−0.143	0.051
	Age				0.098	1.062	−0.083	0.279
	Academic year				−0.016	−0.173	−0.198	0.166
	Academic major				−0.029	−0.601	−0.123	0.065
	Only-child status				0.018	0.348	−0.082	0.118
	Geographic origin				−0.029	−0.574	−0.129	0.071
	Physical activity level				0.179***	3.718	0.085	0.273
	PSQI				−0.190***	−4.049	−0.282	−0.097
	PA × PSQI				−0.147**	−3.169	−0.236	−0.056
Academic procrastination	Gender	0.383	0.147	7.174***	0.007	0.155	−0.088	0.102
	Age				0.233*	2.571	0.054	0.411
	Academic year				−0.273**	−3.004	−0.451	−0.093
	Academic major				−0.022	−0.478	−0.114	0.070
	Only-child status				0.051	1.024	−0.047	0.149
	Geographic origin				−0.003	−0.062	−0.101	0.095
	Physical activity level				−0.117*	−2.452	−0.210	−0.023
	PSQI				0.181***	3.873	0.087	0.275
	Self-efficacy				−0.178***	−3.725	−0.272	−0.084
	PA × PSQI				0.147**	3.192	0.058	0.236

*p < 0.05; **p < 0.01; ***p < 0.001. *β* denotes the standardized regression coefficient, and all 95% confidence intervals are reported on the *β* scale.

Similarly, when academic procrastination was the dependent variable, the overall model was significant (*R*^2^ = 0.147, *F* = 7.174, *p* < 0.001). Older age was concurrently associated with higher procrastination scores (*β* = 0.233, *t* = 2.571, *p* < 0.05), whereas more advanced grade level was associated with lower procrastination scores (*β* = −0.273, *t* = −3.004, *p* < 0.01). Physical activity level was significantly negatively associated with academic procrastination (*β* = −0.117, *t* = −2.452, *p* < 0.05, 95% CI [−0.210, −0.023]), while the PSQI was positively associated with academic procrastination (*β* = 0.181, *t* = 3.873, *p* < 0.001, 95% CI [0.087, 0.275]). Self-efficacy also demonstrated a robust negative association with academic procrastination (*β* = −0.178, *t* = −3.725, *p* < 0.001, 95% CI [−0.272, −0.084]). Most notably, the interaction between physical activity level and the PSQI was significantly and positively associated with academic procrastination (*β* = 0.147, *t* = 3.192, *p* < 0.01, 95% CI [0.058, 0.236]). This result confirms that sleep quality moderated the direct concurrent association between physical activity and academic procrastination: the inverse relationship between physical activity and academic procrastination was more pronounced at better sleep levels and weakened as sleep quality deteriorated.

#### 4.6.2 Conditional indirect effects

Consistent with the interaction effects above, the indirect effect of physical activity on academic procrastination via self-efficacy varied significantly across levels of sleep quality (Table 6 and Figure 3). At low PSQI levels (−1 SD, reflecting better sleep quality), the standardized conditional indirect effect was statistically significant (*β* = −0.058, Boot SE = 0.023, 95% CI [−0.107, −0.019]). At the mean PSQI level, the indirect effect remained statistically significant (*β* = −0.032, Boot SE = 0.013, 95% CI [−0.060, −0.010]). However, at high PSQI levels (+1 SD, reflecting poorer sleep quality), the conditional indirect effect was no longer distinguishable from zero (*β* = −0.005, Boot SE = 0.015, 95% CI [−0.032, 0.024]).

**Table 6:** Conditional indirect effects and the index of moderated mediation at different PSQI levels.

Moderator	Standardized conditional indirect effect				Standardized index of moderated mediation			
	*β*	Boot SE	95% CI for *β* LLCI	95% CI for *β* ULCI	Standardized index	Boot SE	95% CI LLCI	95% CI ULCI
Low PSQI	−0.058	0.023	−0.107	−0.019	0.027	0.011	0.005	0.059
Mean PSQI	−0.032	0.013	−0.060	−0.010				
High PSQI	−0.005	0.015	−0.032	0.024				

Bootstrapped 95% confidence intervals based on 5,000 resamples. If the CI excludes zero, the effect is significant at *α* = 0.05.

**Figure 3:**
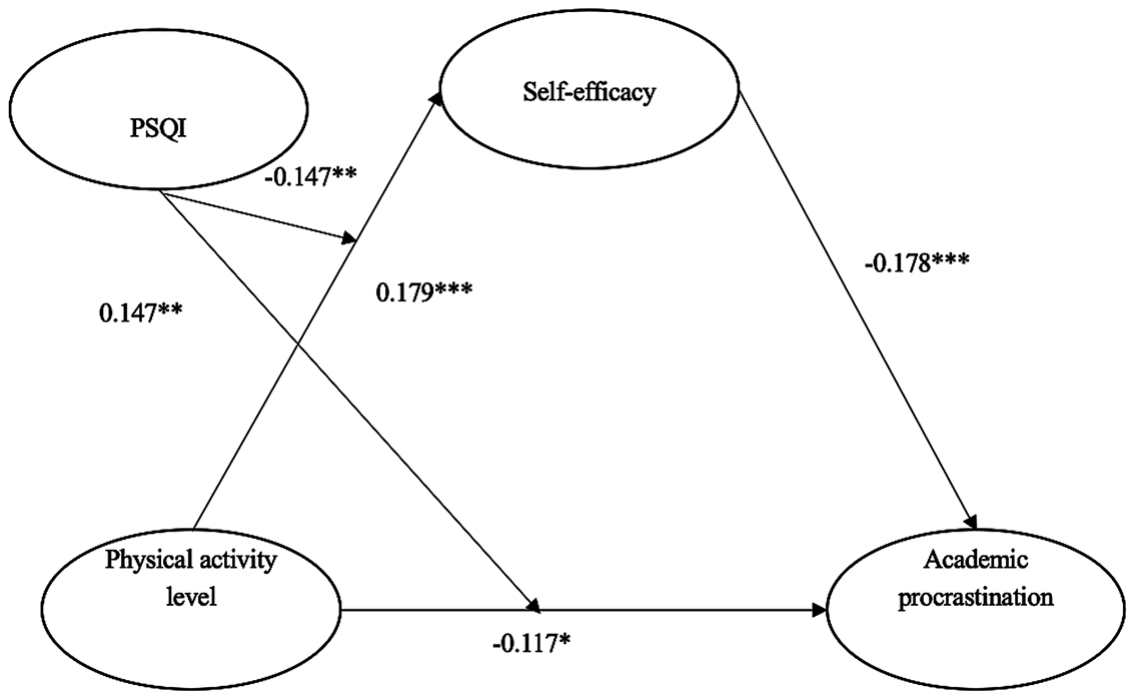
Results of the moderated mediation model of physical activity level and academic procrastination with PSQI as the moderator. (*p < 0.05; **p < 0.01; ***p < 0.001).

The standardized index of moderated mediation was 0.027 (boot SE = 0.011, 95% CI [0.005, 0.059]), with the confidence interval excluding zero. This indicates that sleep quality significantly moderated the strength of the indirect association between physical activity and academic procrastination through self-efficacy. Collectively, among individuals with better sleep quality, the association between physical activity and academic procrastination was more pronounced, operating via self-efficacy. Conversely, this indirect association was attenuated among individuals with poorer sleep quality.

#### 4.6.3 Conditional direct effects at different PSQI levels

To further elucidate the moderating role of sleep quality in the direct pathway, the conditional direct effects of physical activity on academic procrastination were computed at low, mean, and high PSQI levels (±1 SD) (Table 7 and Figure 3). When the PSQI was at a low level (−1 SD, better sleep quality), the direct effect of physical activity on academic procrastination was significant and negative (*β* = −0.164, Boot SE = 0.041, 95% CI [−0.245, −0.082]). At the mean PSQI level, the direct effect remained statistically significant (*β* = −0.072, Boot SE = 0.029, 95% CI [−0.129, −0.014]). In contrast, at high PSQI levels (+1 SD, poorer sleep quality), the direct effect was no longer statistically significant (*β* = +0.020, Boot SE = 0.041, 95% CI [−0.060, 0.100]).

**Table 7:** Conditional direct effects of physical activity level on academic procrastination at different PSQI levels.

Moderator	*β*	Boot SE	95% boot CI LLCI	95% boot CI ULCI
Low PSQI	−0.164	0.041	−0.245	−0.082
Mean PSQI	−0.072	0.029	−0.129	−0.014
High PSQI	0.020	0.041	−0.060	0.100

Collectively, these conditional estimates delineate a clear gradient pattern whereby the inverse association concurrently observed between physical activity and academic procrastination attenuates progressively as sleep quality worsens. This pattern parallels the moderating trend identified in the first-stage indirect pathway (Table 6), jointly supporting the role of sleep quality as a boundary condition for the observed associations between physical activity and academic procrastination (Figure 3).

## 5 Discussion

The present study examined the relationship between physical activity and academic procrastination among college students, with a particular focus on the mediating role of self-efficacy and the moderating role of sleep quality. By integrating these two mechanisms into a single moderated mediation framework, this study provides the first empirical evidence that sleep quality serves as a boundary condition governing both the direct and indirect pathways through which physical activity relates to procrastination. Consistent with our hypotheses, the results revealed that physical activity was not only directly and negatively associated with academic procrastination but also indirectly associated with procrastination through self-efficacy. Furthermore, sleep quality significantly moderated both the first-stage path (physical activity → self-efficacy) and the direct path (physical activity → academic procrastination). Specifically, the beneficial effects of physical activity on self-efficacy and procrastination were amplified under conditions of good sleep quality but attenuated or nullified under poor sleep quality. These findings contribute to the growing literature on the psychological mechanisms linking physical activity to academic outcomes and highlight the importance of considering sleep as a critical contextual factor.

### 5.1 Mediation of self-efficacy

The mediation analysis revealed that self-efficacy partially mediated the relationship between physical activity and academic procrastination, with the indirect effect accounting for a notable portion of the total effect. Consistent with social cognitive theory, physical activity appears to serve as a source of mastery experiences, thereby enhancing students’ beliefs in their capabilities. This heightened self-efficacy, in turn, fosters greater perseverance and self-regulation in academic tasks, reducing the likelihood of procrastination.

However, it is noteworthy that the effect sizes observed in this study were modest. The standardized effect of physical activity on academic procrastination fell within the small-to-medium range, as did the effect on self-efficacy, and the variance explained in the two primary regression models was relatively small. Benchmarked against Cohen’s (1988) conventions for behavioral science—where a *β* of approximately 0.10 represents a small effect and *R*^2^ values of 0.01, 0.06, and 0.14 correspond to small, medium, and large effects, respectively—our observed coefficients fall within the small-to-medium range. This aligns with prior observational research in this domain (Kuai et al., 2025; Zhuan et al., 2024). It is crucial to recognize that small effect sizes are the norm rather than the exception in psychological research involving multifaceted outcomes such as procrastination. Critically, from a clinical perspective, a *β* value of −0.127 indicates that increasing physical activity by one standard deviation is associated with a noticeable decrease in procrastination tendencies. Given that procrastination is a chronic issue for nearly 50% of college students (Kuai et al., 2025; Li et al., 2022), even this modest shift at the individual level can translate into meaningful improvements in academic functioning and mental wellbeing.

Notably, our direct effect was slightly smaller than that reported by Ren et al. (2021) in a comparable Chinese college sample. This discrepancy likely stems from our inclusion of six additional covariates (e.g., academic year and only-child status), which typically attenuates effect sizes and yields more conservative estimates less prone to omitted variable bias. It is imperative to note that Cohen’s benchmarks are heuristic guidelines rather than absolute criteria for substantive significance; therefore, the practical utility of an effect must be evaluated within its real-world context. The modest total effect observed in the full sample should not be misinterpreted as an inherently weak association, but rather as a reflection of subgroup heterogeneity, which we elaborate on in the moderation analysis below.

The modest proportion of variance explained by self-efficacy is theoretically plausible for several reasons. First, prior research suggests that self-control and self-efficacy act as parallel mediators in the physical activity–procrastination relationship (Li et al., 2022), while body self-esteem and overall self-esteem function as serial mediators (Zhuan et al., 2024). This implies that self-efficacy shares explanatory power with multiple psychological constructs rather than acting as the sole mechanism. Second, the cross-sectional design relying entirely on self-reported measures is susceptible to common method variance, which can attenuate observed effect sizes (Podsakoff et al., 2003). Third, academic procrastination is a multiply determined outcome influenced by personality traits (e.g., conscientiousness), academic motivation, and task characteristics (Steel, 2007). Consequently, the modest mediation proportion reflects the etiological complexity of procrastination.

### 5.2 Moderation of sleep quality and theoretical implications

A key contribution of this study is the identification of sleep quality as a significant moderator across multiple pathways. Moderated mediation analyses indicated that the positive association between physical activity and self-efficacy, as well as the negative association between physical activity and procrastination, was significant only for students with average or good sleep quality, but not for those with poor sleep quality. This moderation effect is central to understanding the modest total effect: the association is robust among well-slept students but null among poor sleepers, effectively suppressing the overall estimate. This finding extends previous research by Kuai et al. (2025) and positions sleep quality as a critical boundary condition. Interestingly, our finding that sleep quality moderates the physical activity → self-efficacy pathway contrasts with Wang G. et al. (2026), who identified sleep quality as a mediator. This divergence may stem from differences in covariate adjustment (e.g., controlling for anxiety) or sampling variability (e.g., differing proportions of students with poor sleep quality). Rather than viewing these findings as contradictory, they may reflect different stages of the underlying process: Wang G. et al. (2026) highlight the mechanistic pathway (sleep as a consequence of activity), whereas our data underscore the conditional necessity of sleep as a foundational resource. This suggests that adequate sleep may not just be an outcome of healthy behaviors but a prerequisite for the cognitive appraisal processes necessary to convert physical activity into self-efficacy.

From a conservation of resources theory perspective, adequate sleep may be positively associated with the consolidation of mastery experiences derived from physical activity into generalized self-efficacy beliefs. Sufficient sleep is related to executive function and emotional regulation capacity, which may help students to fully appraise their exercise achievements and maintain their sense of competence. Conversely, poor sleep is associated with reduced cognitive resources necessary for this appraisal process, thereby attenuating the positive association between physical activity and self-efficacy. While our cross-sectional data preclude definitive causal inference regarding this directional pathway, this interpretation is consistent with emerging evidence that sleep supports the translation of mastery experiences into self-efficacy (Wang G. et al., 2026). Physiologically, sufficient sleep promotes neuroendocrine recovery and metabolic homeostasis (Nobari et al., 2023), whereas sleep insufficiency may elevate systemic inflammation and compromise prefrontal cortex function—factors that indirectly undermine the psychological benefits of physical activity (Hatia et al., 2024). Future experimental studies are warranted to validate these proposed physiological and cognitive mechanisms.

### 5.3 Limitations and future directions

This study explored the relationships between physical activity, self-efficacy, sleep quality, and academic procrastination using a cross-sectional design. However, several limitations should be addressed in future research. First, the cross-sectional design is the primary limitation of this study, as it precludes definitive causal inferences. Critically, this design cannot establish temporal precedence—i.e., whether physical activity precedes reductions in procrastination or whether students with lower baseline procrastination are more likely to engage in regular physical activity—leaving causal direction ambiguous. While our findings reveal significant associations and potential pathways, they cannot strictly establish causality. For instance, the relationships among these variables may be reciprocal or influenced by other unmeasured factors. Future research should adopt longitudinal or experimental designs (e.g., randomized physical activity interventions) to examine causal directions and dynamic processes more rigorously.

Second, the current study adjusted for only a limited set of covariates (gender, age, academic major, academic year, only-child status, and geographic origin) in regression models. Several well-documented potential confounders of the physical activity–academic procrastination association were not measured or controlled for, including academic performance (GPA), symptoms of anxiety and depression, socioeconomic status, daily recreational screen time, body mass index (BMI), and objective sleep duration. For example, students with higher GPA may be more likely to engage in regular physical activity and less likely to procrastinate; similarly, elevated anxiety/depression symptoms are associated with both lower physical activity levels and higher procrastination, which could bias the observed associations if unadjusted. Future studies should collect comprehensive covariate data to minimize residual confounding and provide more precise estimates of the relationships tested here.

Third, the reliance on self-reported data raises concerns regarding common method variance, recall bias (e.g., inaccurate estimation of exercise frequency), and social desirability bias (e.g., underreporting procrastination). Although procedural remedies (e.g., anonymity and reverse-scored items) were implemented, and both Harman’s single-factor test and the full collinearity VIF analysis indicated no severe bias, the potential for inflated correlations remains. Consequently, the observed associations may be partially attributable to shared method variance rather than solely reflecting substantive theoretical relationships. To enhance objectivity and causal inference, future studies should adopt multi-source designs, integrating objective measures such as accelerometers and smartwatches to monitor physical activity and sleep physiologically alongside self-report instruments.

Fourth, the study population consists solely of university students from Shanghai—a municipality in eastern China with distinct socioeconomic, cultural, and higher education resource characteristics compared to other regions of the country. Consequently, the findings may not generalize to student populations in other geographic regions (e.g., central and western China) or to institutions with markedly different profiles (e.g., private universities and institutions serving predominantly local students). Additionally, our online recruitment strategy may have introduced selection bias: students who voluntarily completed the survey may differ systematically from non-participants (e.g., being more health conscious, having higher digital literacy, or experiencing less severe procrastination), potentially limiting representativeness even within Shanghai universities. Future multi-site studies employing strict probability sampling techniques across a wider range of geographic regions and institutional types are needed to validate and extend our results.

Finally, the exploration of internal mechanisms is limited. This study preliminarily examined the mediation of self-efficacy and the moderation of sleep quality but did not delve deeply into how sleep quality affects these psychological processes. For example, poor sleep may impair cognitive control, emotional regulation, or neuroendocrine functions, thereby weakening the psychological benefits of physical activity. Future research could incorporate cognitive neuroscience techniques (e.g., ERP and functional magnetic resonance imaging [fMRI]) or measure physiological indicators such as stress hormones and inflammatory markers to reveal the underlying biological and cognitive mechanisms.

### 5.4 Practical implications and public health significance

The clinical relevance of these findings is further underscored by the moderation analysis. While physical activity alone appeared insufficient to reduce procrastination among poor sleepers in our data, it showed a moderate association among students with adequate sleep. Despite the modest individual-level effect sizes in the full sample, the public health implications of this study are considerable. Given that approximately half of college students experience chronic academic procrastination (Kuai et al., 2025; Li et al., 2022), even small shifts in physical activity patterns are associated with substantial population-level benefits. Isotemporal substitution analyses suggest that replacing 15–20 min of sedentary behavior with moderate-to-vigorous physical activity is associated with lower procrastination scores (Kuai et al., 2025). Extrapolated to a university-wide scale, such low-threshold changes represent a highly cost-effective strategy for improving academic engagement.

Crucially, our moderation analysis reveals that the total effect observed in the full sample masks significant subgroup heterogeneity. For students with good or average sleep quality, the association between physical activity and procrastination was considerably larger. This pattern tentatively suggests that, for the “good sleeper” subgroup, physical activity may serve as a useful component in managing procrastination. However, given the cross-sectional nature of our data, these associations should not be interpreted as causal evidence, and any practical recommendations must be considered preliminary. Psychologists and university counseling services might consider promoting accessible, low-cost physical activity options (e.g., campus walking challenges) coupled with self-efficacy enhancement strategies, such as goal-setting and progress monitoring, while remaining mindful that these suggestions are based on correlational evidence. Conversely, for students reporting poor sleep quality, our data suggest that physical activity alone may not be sufficient to mitigate procrastination. Practitioners should be cautious about recommending standalone physical activity interventions for this subgroup; instead, such efforts might benefit from being combined with sleep hygiene interventions (e.g., stimulus control and circadian alignment). As a tentative implication, a differentiated approach—focusing on physical activity and self-efficacy for good sleepers and integrating activity with sleep interventions for poor sleepers—could be considered by mental health professionals when designing wellness programs, pending confirmation from experimental or longitudinal research.

## 6 Conclusion

This study, through a moderated mediation model, reveals associations between physical activity and college students’ academic procrastination, as well as boundary conditions for these associations. Cross-sectional findings indicate that physical activity is associated with lower academic procrastination, with self-efficacy accounting for a portion of this observed association. More importantly, sleep quality plays a crucial moderating role in this process: good sleep quality is associated with a stronger negative association between physical activity and procrastination and a stronger positive association between physical activity and self-efficacy, while poor sleep quality is associated with weakened links. While these findings cannot establish causality, they suggest that physical activity, self-efficacy, and sleep quality are synergistic factors relevant to understanding academic procrastination among college students. The results provide preliminary empirical support for the future development of integrated intervention strategies targeting these modifiable factors, although the effectiveness of such interventions requires validation in experimental studies. Notably, the finding that the associations between physical activity with self-efficacy and procrastination were only evident among students with adequate sleep quality highlights a previously unrecognized contingency: the potential benefits of physical activity may depend on the co-occurrence of good sleep. This insight extends prior literature by moving beyond main-effect approaches to identify for whom and under what conditions physical activity is most strongly linked to reduced procrastination, thereby informing more targeted, stratified intervention designs.

## Data Availability

The raw data supporting the conclusions of this article will be made available by the authors, without undue reservation.
